# Blink rate is associated with drug-induced parkinsonism in patients with severe mental illness, but does not meet requirements to serve as a clinical test: the Curacao extrapyramidal syndromes study XIII

**DOI:** 10.1186/s12952-017-0079-y

**Published:** 2017-08-25

**Authors:** Charlotte L. Mentzel, P. Roberto Bakker, Jim van Os, Marjan Drukker, Glenn E. Matroos, Marina A. J. Tijssen, Peter N. van Harten

**Affiliations:** 1 Department of Psychiatry and Psychology, Maastricht University Medical Centre, South Limburg Mental Health and Teaching Network, Maastricht, The Netherlands; 2 Psychiatric Centre GGZ Centraal, Utrechtseweg 266, 3818 EW Amersfoort, The Netherlands; 3 King’s College London, King’s Health Partners, Department of Psychosis Studies, Institute of Psychiatry, London, UK; 4 Psychiatric Centre GGz Curaçao, Groot Kwartier, Curaçao; 5 Department of Neurology, University Medical Centre Groningen (UMCG), University of Groningen, Groningen, the Netherlands

**Keywords:** Drug-induced parkinsonism, Severe mental illness, Antipsychotic, Blink rate

## Abstract

**Background:**

Drug-induced parkinsonism (DIP) has a high prevalence and is associated with poorer quality of life. To find a practical clinical tool to assess DIP in patients with severe mental illness (SMI), the association between blink rate and drug-induced parkinsonism (DIP) was assessed.

**Methods:**

In a cohort of 204 SMI patients receiving care from the only mental health service of the previous Dutch Antilles, blink rate per minute during conversation was assessed by an additional trained movement disorder specialist. DIP was rated on the Unified Parkinson’s Disease Rating Scale (UPDRS) in 878 assessments over a period of 18 years. Diagnostic values of blink rate were calculated.

**Results:**

DIP prevalence was 36%, average blink rate was 14 (standard deviation (SD) 11) for patients with DIP, and 19 (SD 14) for patients without. There was a significant association between blink rate and DIP (*p* < 0.001). With a blink rate cut-off of 20 blinks per minute, sensitivity was 77% and specificity was 38%. A 10% percentile cut-off model resulted in an area under the ROC curve of 0.61. A logistic prediction model between dichotomous DIP and continuous blink rate per minute an area under the ROC curve of 0.70.

**Conclusions:**

There is a significant association between blink rate and DIP as diagnosed on the UPDRS. However, blink rate sensitivity and specificity with regard to DIP are too low to replace clinical rating scales in routine psychiatric practice.

**Trial registration:**

The study was started over 20 years ago in 1992, at the time registering a trial was not common practice, therefore the study was never registered.

## Background

Drug-induced parkinsonism (DIP) prevalence in patients with severe mental illness (SMI) varies between 36% [1] and 56% [2], and is associated with a poorer quality of life [3], falls [4] and antipsychotic non-compliance [5]. However, DIP is poorly recognised and both DIP and Parkinson’s disease (PD) rating scales requiring lengthy training sessions are difficult to implement, hence rating scales are not suitable for clinical practice [6]. Therefore, simple and easy to use diagnostic methods for DIP are warranted. Diagnostic methods based on blink rate as a clinical test for diagnosing DIP would be a good measure because: (i) the assessment of blink rate during conversation is easy and quick, (ii) requires no specialised equipment, (iii) has a high interrater reliability [7],, and (iv) research in PD has shown that reduced blink rate during conversation discriminates well between PD and healthy controls when compared to the Unified Parkinson’s Disease Rating Scale (UPDRS) [8]. Blink rate may be easily measured with the use of mobile apps, thus enabling clinicians to diagnose DIP.

D2-receptor involvement has been linked consistently to spontaneous blink rate, both in human and animal experiments [9]. In their 1990 seminal paper, Karson et al. [7] conclude that blinks are most likely generated in the pontine reticular formation and signals are then transmitted to the lateral geniculate bodies. Since this publication, to our knowledge, only three articles examining blink rate in schizophrenia have been published. These studies linked blink rate to various neurological soft signs (NSS) in patients [10–12] however no association between blink rate and central dopaminergic activity was found in healthy controls [9]. While several studies indicate that blink rate is a good clinical test for the diagnosis of PD, as far as we know, no such study has been published on the use of blink rate as a clinical test for DIP in patients with severe mental illness (SMI).

The present paper aims to assess (i) the association between DIP and blink rate, and (ii) the possibilities of using blink rate as a clinical test to diagnose DIP with the UPDRS [1, 13] as gold standard. As the goal of the paper is to develop a clinical test to differentiate between SMI patients with DIP and SMI patients without DIP, patients were compared to other patients and no healthy control group was used.

## Methods

### Subjects

All 204 patients hospitalized or receiving structured outpatient care from the Dr. D.R. Capriles Clinic, the only psychiatric hospital in the Dutch Antilles, in 1991 were asked to take part in the Curacao extra pyramidal syndromes study, an 18 year (1993, 1994, 1996, 1997, 1998, 2001 and 2009) prospective naturalistic follow-up study. Informed consent was obtained from all patients and the protocol was approved by the Curaçao Institutional Review Board. A total of 8 assessments focusing on movement disorders and medication use were performed over the 18 years follow-up. A detailed description of the patients and assessments has been published previously [1].

Inclusion criteria were a minimum age of 18 years, and cumulative exposure to antipsychotics of at least 3 months; current antipsychotic use was not required. Exclusion criteria were a history of neurological disorders affecting motor function, including PD, and having undergone a lobotomy. Patients with dementia (*N* = 7) or mental retardation (*N* = 3) as primary diagnosis were excluded. The total number of patients was 191 and the dataset is available from the corresponding author upon request.

### Assessments

The UPDRS version 3.0 was used to define DIP [13]. Blink rate per minute was assessed for 1 min during conversation at each measurement (*N* = 878) by one author with a stopwatch while the other author conducted the interview (PvH and GM), a more detailed description of the test has been published previously [1]. Both raters are psychiatrists specialized in movement disorders. They were blind to the UPDRS score while blink rate was being assessed and vice versa. An exact description of the test circumstances can be found in a previous publication [1]. DIP was defined as (i) a score of at least ‘moderate’ (score 3, range 0–4) on one of the bradykinesia items (1, [2], 6–14) or two or more scores of ‘mild’ (score 2) on these items; (ii) a rigidity (item 3) or tremor (item 4–5) score of at least ‘mild’. The more stringent criteria used for bradykinesia were chosen as motor slowness can also be caused by mental symptoms or medication. Time points on which the patient scored ‘mild’ or higher on the blepharospasm item on the Burke Fahn Marsden Dystonia Rating Scale (BFMDRS) were excluded (*N* = 54), as blepharospasm can cause involuntary contractions in the eyelid and hence can be misclassified as blink-related DIP. The BFMDRS was also scored at all of the time points by the same raters (PvH and GM). DSM-III-R diagnosis and demographic variables (age, sex, diagnosis, and antipsychotic type and dose), were extracted from the case file by a trained physician.

### Statistical analyses

Analyses were carried out with Stata, version 12 [14]. Blink rate per minute was (i) used as a continuous variable, (ii) dichotomised using a cut-off point of 20 blinks per minute, as suggested by Fitzpatrick et al. [8], and (iii) as 10% percentile cut-offs (hereafter: continuous and dichotomous blink rate, and 10% percentile blink rates, respectively). Using both dichotomous blink rate and 10% percentile blink rates we calculated: (i) sensitivity and specificity using the *roctab* (nonparametric ROC analysis)command; (ii) positive predictive value (PPV) and negative predictive value (NPV) using the *diagt* (summary statistics for diagnostic tests compared to true disease status) command. The association between blink rate and DIP as a continuous variable was calculated using the *regress (linear regression)* command. Areas under the ROC curves were calculated: (i) for the 10% percentile blink rate using the roctab command, and (ii) for the continuous blink rate per minute using the *lroc* (computes area under ROC curve and graph the curve)command based on the logistic prediction model using the *logit(logistic regression, reporting coefficients)* command, with DIP as a dichotomous dependent variable and continuous blink rate, age, sex, diagnosis (schizophrenia or other) and antipsychotic defined daily dose (DDD) [15] and type as independent variables.

## Results

A total of 878 assessments in 191 patients were available for analysis. All patients provided written informed consent. Of the sample, 72% was male, 95% was of African-Caribbean origin and 84% had a DSM-III-R diagnosis of schizophrenia. The mean age was 53 years with a standard deviation (SD) of 15 years, DIP prevalence according to the UPDRS was 36% (317 instances in 890 measurements, mean severity 20 points on the UPDRS, SD 12), DIP persisted to the next time point in 65% of cases. Mean blink rate was 14 (SD 11) for patients with DIP, and 19 (SD 14) for patients without DIP.

**Fig. 1:**
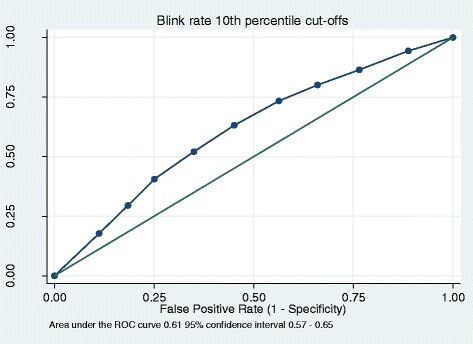
Receiver operated curves (ROC) for blink rate as a diagnostic tool for drug-induced parkinsonism using 10th percentile cut offs

**Fig. 2:**
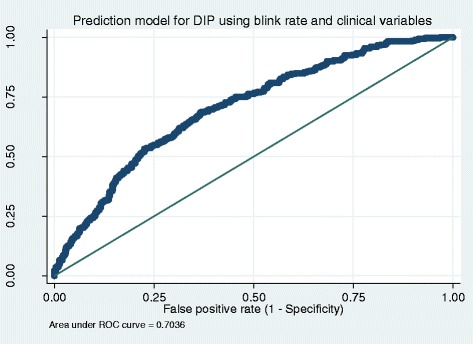
Receiver operated curves (ROC) constructed from the prediction model for drug-induced parkinsonism using blink rate and covariates

For the dichotomous blink rate, sensitivity (the test’s ability to correctly designate a subject with the disease as positive) was 77%, specificity (the test’s ability to correctly designate a subject without the disease as negative) 38%, PPV 75% (meaning there is a 75% probability that if a patient’s blink rate is below 20 blinks per minute that patient does indeed have DIP), and NPV 41% (meaning there is a 41% probability that if a patients blink rate is higher than 20 blinks per minute, the patient does not have DIP) (Table 1). For the 10% percentile blink rates sensitivity, specificity, PPV and NPV are reported in Table 2. The area under the ROC curve was 0.61 (Fig. 1). Linear regression yielded significant coefficients between DIP and blink rate (B − 0.14, *p* < 0.000) with an R-squared of explained variance of 0.025 or 2.5%. In the logistic regression prediction model adjusted for age, sex, diagnosis, and antipsychotic type and dose, R-squared explained variance was marginally higher at 0.095 or 9.5% (Fig. 2 and Table 3). The ROC derived from the prediction model yielded an area under the curve of 0.70, slightly higher than the ROC of the 10th percentile cut-off.

**Table 1:** Drug-induced parkinsonism identified by the UPDRS as gold standard, based on blink rate

Parkinsonism			
UPDRS	Blink rate no case^a^	Blink rate case	Total
No case	216	347	563
Case	74	241	315
total	290	588	878
	Sensitivity: 77%, Specificity: 38%		
	Positive predictive value 75%, Negative predictive value 41%		

^a^The cut-off for a case is 20 blinks per minute

**Table 2:** Sensitivity, specificity and positive and negative predictive value using a 10TH percentile cut off

Percentile	N	Blink rate per minute	Sensitivity	Specificity	Correctly Classified	PPV	NPV
10th	101	37-87	100.00%	0.00%	36.98%	22%	63%
9th	94	28-36	92.47%	10.98%	41-.11%	25%	62%
8th	97	23-27	85.71%	21.34%	45.15%	25%	60%
7th	120	18-22	76.88%	30.95%	47.93%	26%	58%
6th	108	15-17	68.05%	44.05%	52.93%	27%	57%
5th	65	13-14	58.70%	55.03%	56.39%	29%	56%
4th	105	10-12	52.21%	61.13%	57.83%	31%	54%
3rd	131	7-9	43.12%	71.80%	61.19%	33%	55%
2nd	88	5-6	27.27%	82.47%	62.06%	34%	56%
1st	132	0-4	16.10%	89.33%	62.25%	35%	83%
base		0	0.00%	100.00%	63.02%

**Table 3:** Prediction model for drug-induced parkinsonism using covariates and continuous blink rate

	Odds Ratio	*p*-value	95% Confidence interval	
Blink rate per minute	0.97	<0.0000	0.96	0.98
Age	1.04	<0.000	1.03	1.05
Female sex	0.64	0.03	0.44	0.95
Diagnosis other than schizophrenia	1.73	0.01	1.12	2.65
Antipsychotic DDD	1.00	0.96	0.51	0.87
Antipsychotic type				
Only FGA	1.83	0.07	0.96	3.50
Only SGA	0.62	0.28	0.26	1.47
Both FGA and SGA	1.58	0.36	0.60	4.20

## Discussion

The association between blink rate and DIP and UPDRS score in patients with SMI is highly significant (*p* < 0.000). However, the explained variance of 9.5% of the logistic regression model is too small, and sensitivity and specificity of blink rate are too low for use as a clinical tool in SMI patients. With the most efficient cut-off, only 62% of patients was correctly classified by the blink rate test. Other clinical parameters (that are easily accessible to clinicians) that are known to affect DIP, such as age, diagnosis, and sex, were added to a logistic regression model. The variables displayed significant associations with DIP, however the explained variance was still too low for the model to be useful in a clinical setting.

The present findings are in contrast with results found in PD. Using the same cut off point of 20 blinks per minute, a meta-analysis [8] reported a sensitivity and specificity of 65% and 83%, respectively, whereas the present study found a sensitivity and specificity of 77% and 38%, respectively. A possible cause for the discrepancy is the greater difference in mean blink rate between patients with PD and healthy controls (18 versus 34 blinks) compared to the difference between SMI patients with and without DIP (14 versus 19 blinks) in the present study. An explanation is that patients with PD and healthy controls are distinct groups, without much overlap, whereas the SMI patients in the present study originate from the same population and have DIP on a continuous scale.

In the present study, SMI patients without DIP showed a lower average blink rate per minute compared to healthy controls from other studies with similar methodology. This is surprising as studies have consistently shown that patients with schizophrenia have a higher average blink rate compared to healthy controls [7, 11, 12], with a blink rate of 27 for patients with a psychotic disorder and 22–18 for other mental disorders [7]. Although this difference is most striking in drug-naive patients with a diagnosis of schizophrenia [7, 11, 12, 16], it is also present in patients treated for schizophrenia [10, 12]. Furthermore, blink rate is associated with subsets of symptoms such as hallucinations and anxiety [10–12] in patients with a diagnosis of schizophrenia. Associations with neurological soft signs [10, 11, 16] and antipsychotic dose [16] have been inconsistent. It is likely that blink rate in patients with schizophrenia is influenced by more factors than just DIP. What these factors are and how they relate to the current study population remains unknown. Further investigation into the pathophysiology of abnormal blink rates in patients with a diagnosis of schizophrenia is warranted as it could shed light on underlying disease mechanisms.

### Limitations

Due to the naturalistic setting, the well-defined catchment area and the broad inclusion criteria, results from this study are likely to be a good representation of movement disorders in a real world SMI population; Bakker et al. [17] found very similar results for medication use and movement disorders in a Dutch population However, there are a number of limitations to the study. First, blink rate varies with context and, therefore, also varies between tests. Although no data on inter-rater reliability was available in this study, both Karson et al. [7] and Fitzpartrick et al. [8] reported good inter-rater reliability and test-retest reliability of blink rate assessment during conversation. However, comparisons with blink rate in other studies that use different tests are difficult. Second, the more stringent criteria for bradykinesia used in the current study to diagnose DIP [2] is not in line with the UK brain bank cut-off for PD [18]. However, post-hoc analysis using the UK brain bank cut-off showed highly similar results. Third, the UPDRS is the most common tool to diagnose PD and is much more comprehensive than other scales used to measure DIP [19]. However, a number of experimental instrumental measurements of both PD [20] and drug-induced bradykinesia [21] are likely more accurate than the UPDRS, hence might result in a more accurate diagnosis of DIP and possibly a higher sensitivity and specificity for blink rate. Fourth, repeated measures over time could result in bias if there were differential attrition. However, post-hoc analyses with only one measurement per patient showed very similar results. Finally, Annamalai et al. [22] found an association between smoking and nicotine in their impact on the dopaminergic system. Unfortunately, in the current study, no data on smoking was available. However, it is very unlikely that adding a smoking variable to the logistic regression model would have a substantial impact on the sensitivity en specificity of the test.

## Conclusions

There is a significant association between blink rate and DIP as diagnosed on the UPDRS. Unfortunately, blink rate sensitivity and specificity with regard to these outcomes are too low to replace clinician rating scales in routine practice. However, there is still a need for an easier and more accessible way to diagnose movement disorders in mental health services, as DIP is highly prevalent in SMI patients [1, 2, 19] and negatively impacts quality of life. DIP is currently under-diagnosed [19–21], as clinical rating scales present a number of problems for use in daily clinical practice [20, 21].Therefore, future research and clinical practice into diagnosing DIP may be served by combining blink rate with instrument measurements, e.g. a finger tapping test, a tremor test and/or a reaction speed test. All these measures could be programmed as an app on a mobile device for ease of use. More research needs to be done into the validity of these combinations.

## Abbreviations

BFMDRS: Burke Fahn Marsden Dystonia Rating Scale
DIP: Drug-induced parkinsonism
NPV: Negative predictive value
PD: Parkinson’s disease
PPV: Positive predictive value
SMI: Severe mental illness
UPDRS: Unified Parkinson’s Disease Rating Scale
